# Ability of Arkansas LaKast and LaKast Hybrid Rice Bran to Reduce *Salmonella* Typhimurium in Chicken Cecal Incubations and Effects on Cecal Microbiota

**DOI:** 10.3389/fmicb.2018.00134

**Published:** 2018-02-06

**Authors:** Sun Ae Kim, Peter M. Rubinelli, Si Hong Park, Steven C. Ricke

**Affiliations:** Department of Food Science, Center for Food Safety, University of Arkansas, Fayetteville, AR, United States

**Keywords:** *Salmonella*, poultry, reduction, inhibition, cecum, Arkansas rice, microbiome

## Abstract

The objective of the present study was to evaluate the prebiotic ability of Arkansas (AR) LaKast rice bran cultivars as a feed supplement to reduce *Salmonella* Typhimurium and other gut microbiota. An *in vitro* mixed anaerobic culture system was used to simulate conditions in the chicken ceca. Anaerobic cultures contained feed, cecal contents collected from 2, 4, and 6 weeks of chicken broilers, and with/without AR rice bran (pureline and hybrid). After 24 h pre-incubation, *S.* Typhimurium was inoculated into the anaerobic cultures and surviving *S*. Typhimurium were enumerated during anaerobic incubation up to 48 h. Samples were also collected after 0, 6, 12, 24, 48 h incubation for microbiome analysis with an Illumina MiSeq platform to investigate the changes in bacterial composition. Both pure and hybrid LaKast rice exhibited significant inhibitory effects in all experiments using 2, 4, and 6 weeks ceca but greater bactericidal effects by LaKast rice were observed at 6 weeks compared to 2- and 4-week ceca samples. For samples containing 6-week chicken ceca, the pureline and hybrid rice bran resulted in no viable *S*. Typhimurium and 6.58 log CFU/ml reduction after 48 h, respectively. Adding rice bran also led to changes in the cecal microbiota. LaKast rice bran resulted in more diverse bacterial population than control groups without any rice bran. The lowest abundance of *Proteobacteria* (at phylum level) and *Enterobacteriaceae* (at family and genus level) was exhibited in LaKast pure treated groups followed by LaKast hybrid and control. This may be attributed to a significant reduction of *S*. Typhimurium of the *Enterobacteriaceae* family and *Proteobacteria* phylum. This study suggests the beneficial functionality of LaKast rice brans as biological supplements in feed. The use of rice bran is favorable for both the consumer and the rice industry because of the perception of rice bran as a naturally occurring substance. As an abundant by-product of rice production, its use as a prebiotic in chicken feed may add economic value benefiting both the rice and poultry industries.

## Introduction

*Salmonella* is a prevalent foodborne pathogen causing bacterial gastroenteritis in humans throughout the world and the organism is commonly isolated from various foods and environments, such as poultry products (Hope et al., 2002; Centers for Disease Control Prevention, 2009; Scallan et al., 2011). Pathogenic *Salmonella* is of great concern to the poultry industry thus various attempts to control *Salmonella* and prevent potential foodborne disease have been studied (Foley et al., 2011, 2013; Finstad et al., 2012; Howard et al., 2012). Prebiotics are defined as “a non-digestible ingredient which beneficially influences the host by selectively stimulating the growth and/or activity of one or a limited number of bacteria in the colon” and using prebiotics in feed is an emerging pathogen control method in the poultry industry (Gibson and Roberfroid, 1995; Patterson and Burkholder, 2003; Roberfroid, 2007; Ricke, 2015).

Rice bran is the hard outer layer of rice and an underutilized product of rice milling (Zullaikah et al., 2005). Rice bran is the main by-product of rice and is produced when the bran layer is removed from the rice kernel during the milling, whitening, or polishing process. Rice bran contains a variety of nutrients such as complex carbohydrates, proteins, amino acids, minerals, and vitamins as well as various components that exhibit prebiotic effects by modulating microbiota in the intestine (Ryan et al., 2011; Sheflin et al., 2015). Several studies have revealed potential prebiotic properties by demonstrating reduction of *S*. Typhimurium colonization in the gastrointestinal tract when rice bran was added in the diet (Kim et al., 2012, 2013; Kumar et al., 2012). Our previous study investigated the ability of three rice brans including Calrose, Jasmine, and Red Wells to control *S*. Typhimurium, and reported that Calrose rice bran resulted in a rapid inhibition of *S*. Typhimurium as well as significant changes in microbial populations and metabolites (Rubinelli et al., 2017).

Rice production in Arkansas (AR) ranks as the highest in the United States, accounting for 46.5% of the total production, making it a major crop in the state (Talbert and Burgos, 2007; Wilson et al., 2007). Further assessment of AR LaKast rice components could be beneficial to both the rice and the poultry industries prevalent in AR. However, only limited information is available on the effects of LaKast rice bran as a potential prebiotic. In the present study, the ability of LaKast rice bran to reduce *S.* Typhimurium was investigated in a controlled *in vitro* anaerobic mixed cecal culture to determine the potential of LaKast rice brans as prebiotics. Microbiome analysis was employed to evaluate bacterial compositional changes of LaKast rice bran on microbiota within the chicken ceca.

## Materials and Methods

### Bacterial Strains

A nalidixic acid-resistant (NA^R^) marker strain of *Salmonella* Typhimurium ST97 was kindly provided by Dr. Billy Hargis, University of Arkansas, Fayetteville, AR, United States. A second NA^R^ marker strain used was derived from strain UK-1 (gift of Dr. Young Min Kwon, University of Arkansas) and passaged four times in chicks to increase virulence. The bacterial strains were cultured in sterile tubes containing Luria-Bertani (LB) medium supplemented with 20 μg/ml nalidixic acid at 37°C for 16 h with agitation at 250 rpm.

### Arkansas Rice Bran Cultivars

The LaKast pureline and hybrid rice brans were kindly provided by Dr. Griffiths Atungulu, Department of Food Science, University of Arkansas, Fayetteville, AR, United States. These were derived from rice obtained from local rice producers in Burdette, AR, United States.

### Cecal Contents Preparation

**FIGURE 1:**
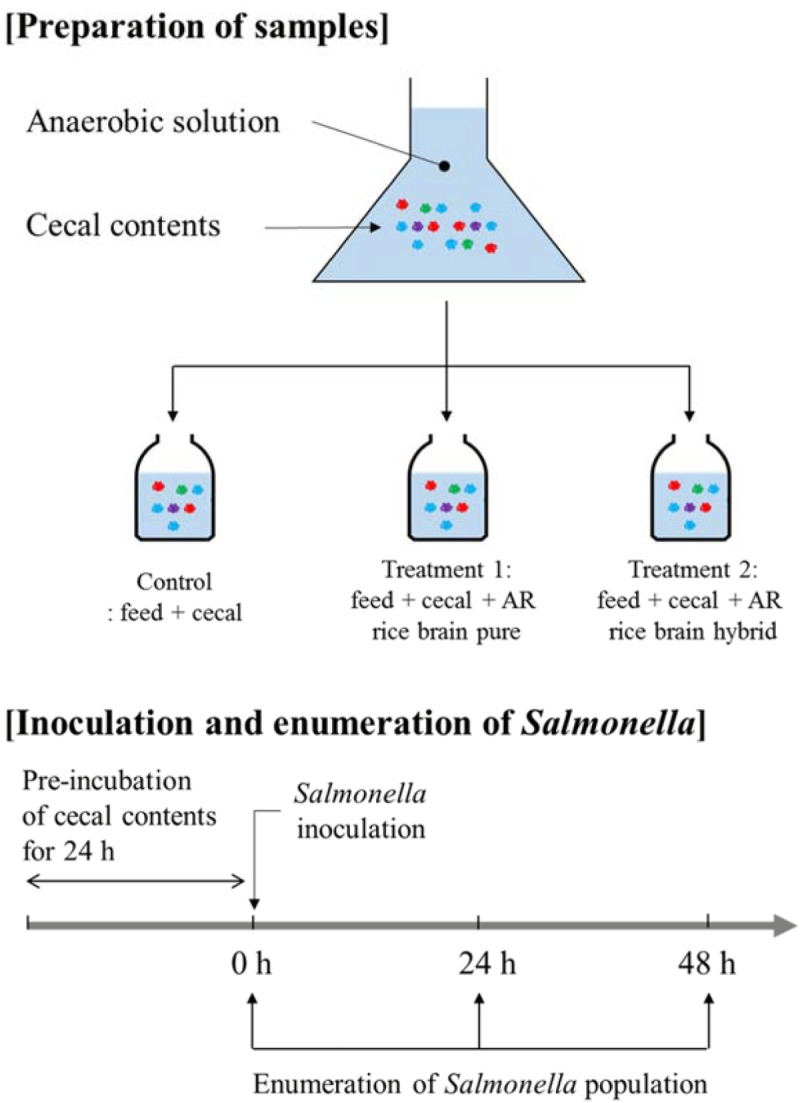
Experimental design in the present study.

The overall experimental design used in the present study is shown in **Figure 1**. Cecal materials used in the present study were obtained from 14-, 28-, and 42-day-old Cobb male broiler chickens (Cobb-Vantress, Inc., Siloam Springs, AR, United States). Chickens were killed by CO_2_ asphyxiation using an approved Institutional Animal Care and Use Committee protocol (IACUC). Ceca were removed aseptically and immediately placed in a sterile sample bag in a portable anaerobic box (Mitsubishi Gas Chemical Co., Japan) containing oxygen-scrubbing sachets. These were subsequently transferred to an anaerobic chamber (Coy Laboratory Products Inc., Grass Lake, MI, United States) containing an atmosphere of 90% nitrogen/5% CO_2_/5% hydrogen.

### Anaerobic *in Vitro* Mixed Cultures

Anaerobic dilution solution was prepared fresh and consisted of 0.45 g/L K_2_HPO_4_, 0.45 g/L KH_2_PO_4_, 0.45 g/L (NH_4_)_2_SO_4_, 0.9 g/L NaCl, 0.1875 g/L MgSO_4_-7H_2_O, 0.12 g/L CaCl_2_-2H_2_O, 1 ml/L 0.1% resazurin, 0.05% cysteine-HCl, and 0.4% CO_2_-saturated sodium carbonate, with the sodium carbonate added last. This medium was used along with chicken feed and chemical fractions of rice bran to culture the anaerobic microorganisms of cecal contents, based on the methods described in previous research (Fan et al., 1995; Shermer et al., 1998; Saengkerdsub et al., 2006; Donalson et al., 2007, 2008; Dunkley et al., 2007; Rubinelli et al., 2016, 2017). Briefly, the anaerobic dilution solution was de-oxygenated using anaerobic gas mixture (90% nitrogen/5% carbon dioxide/5% hydrogen) in an anaerobic chamber for 30 min using an aquarium pump and airstone and subsequently autoclaved. The absence of *S.* Typhimurium in the cecal content was confirmed prior to the experiment by enrichment with tetrathionate (TT) broth (BD Biosciences, San Jose, CA, United States) followed by streaking onto brilliant green agar plates (BD Biosciences).

The cecal content was diluted 1:3000 by addition of 0.1 gram cecal content to 300 ml sterile anaerobic dilution solution. The 20 ml of diluted solution (the anaerobic mixed cultures) was transferred to sterile serum bottles containing 0.25 gram chicken feed as previously described (Fan et al., 1995; Shermer et al., 1998; Saengkerdsub et al., 2006; Donalson et al., 2007, 2008; Dunkley et al., 2007; Rubinelli et al., 2016, 2017). The control group contained only cecal content and feed (no rice bran), and the treatment group contained cecal content, feed, and LaKast rice bran (pure or hybrid) or rice bran fractions were added at a concentration of 1% (w/v). The feed was added to the same concentration used in previous research (Donalson et al., 2007). The anaerobic mixed cultures containing cecal, feed, and/or LaKast rice bran cultivar was pre-incubated at 37°C for 24 h (Model G25, New Brunswick, Inc.,) at 150 rpm. After pre-incubation, culture of *S.* Typhimurium was added to 20 ml culture (approximately 6 log CFU/ml) and incubated at 37°C.

### *Salmonella* Inoculation and Enumeration of Survivors

After 0, 24, and 48 h, an aliquot of each culture was subjected to enumerate *S.* Typhimurium survivors by dilution with sterile phosphate buffer and spread plating onto 2 brilliant green agar plates supplemented with 20 μg/ml NA. If there were no *S.* Typhimurium at a particular time point in the undiluted culture, that culture was enriched with TT and streaked on brilliant green agar plates in order to confirm that no *S.* Typhimurium survived.

### Microbiome Analysis

For the microbiome analysis, the *in vitro* anaerobic mixed cultures containing purline LaKast rice bran, hybrid LaKast rice bran, and “no rice bran” control (feed + cecal control) were collected at various time points (0, 6, 12, 24, and 48 h). Three biological replicate cecal contents were sampled from chickens of ages 2, 4, and 6 weeks old. These were sequenced as 3 technical replicates, for a total of 135 sequenced samples. Genomic DNA was extracted from the cecal contents via a QIAamp Fast DNA Stool Mini Kit following the manufacturer’s instructions (Qiagen, Valencia, CA, United States). Microbiome sequencing was conducted using an Illumina MiSeq platform (Illumina, San Diego, CA, United States), targeting the V4 hypervariable region of 16S rRNA and the resulting sequence reads were analyzed with the Quantitative Insights into Microbial Ecology (QIIME) pipeline (version 1.9.0) as described previously (Park et al., 2016; Kim et al., 2017a).

### Statistical Analysis

The log CFU/ml was determined by averaging all biological replicates and evaluated the general linear model procedure in SAS (version 9.13; SAS Institute Inc., Cary, NC, United States). If analysis of variance (ANOVA) detected a significant result (*P* < 0.05), the mean values were tested using Tukey’s multiple-range test.

## Results and Discussion

Recently, our laboratory reported on the activity of rice bran from different cultivars to reduce *Salmonella* using an *in vitro* mixed anaerobic culture system (Rubinelli et al., 2017). The ceca are the main site of *Salmonella* colonization and it contains a wide range of anaerobic bacteria (Salanitro et al., 1974; Hudault et al., 1985; Fan et al., 1995; Ricke and Pillai, 1999). However, when we collected cecal contents from different ages of broilers and employed an anaerobic mixed culture system to evaluate prebiotic-like effects of a commercial prebiotic, we observed differences in responses related to age of the bird (Rubinelli et al., 2016; Park et al., 2017). The previous experimental approach provides insight on cecal microbial development as a function of bird age (Park et al., 2017) thus the same incremental ages of birds were also employed in this study as sources of inocula.

In our previous study, two different experimental designs were employed; unadapted and adapted incubation (Rubinelli et al., 2017). In the unadapted incubation, the bacteria were inoculated at the beginning of the incubation along with feed, agents, and cecal bacteria. For the adapted incubation, the bacteria were added after pre-incubation for 24 h of the cecal bacteria. Park et al. (2017) observed that the inhibitory effects of Calrose rice bran against *S.* Typhimurium were significantly higher in the adapted condition; suggesting cecal microbiota play an important role to the reduction of *S.* Typhimurium. Further, the adapted condition simulates exposure of pathogenic bacteria after cecal bacteria are metabolically active, similar to the *in vivo* conditions. Thus, the adapted experimental condition was used exclusively in the present study.

**FIGURE 2:**
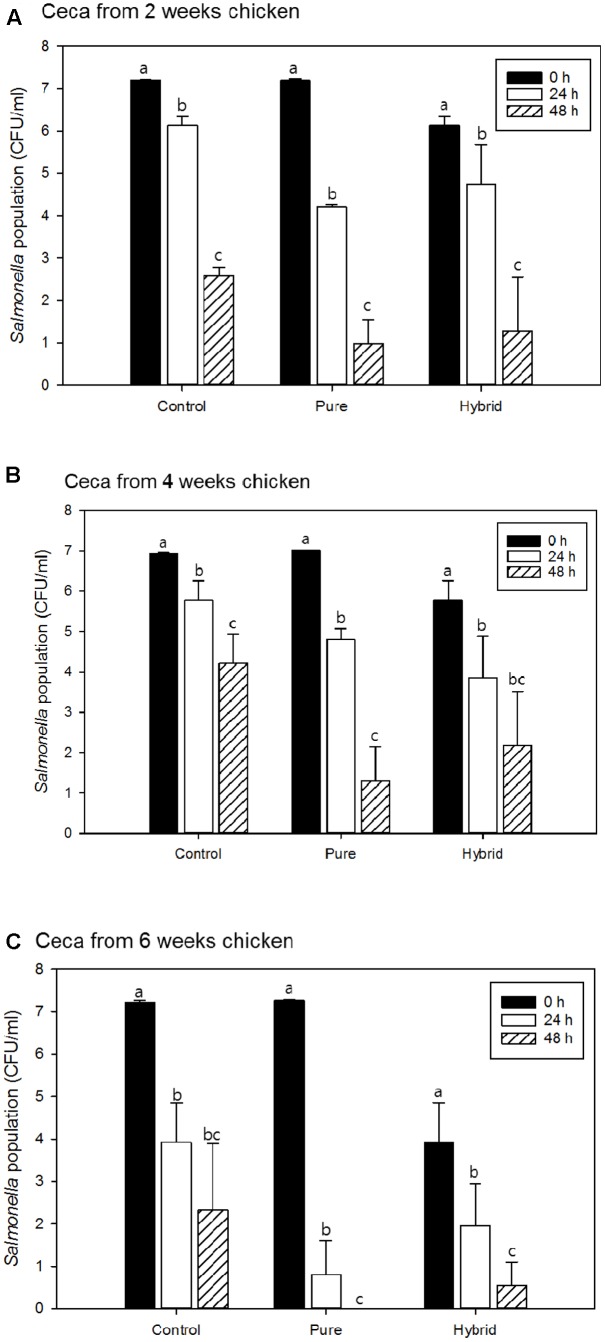
*Salmonella* Typhimurium population of control and treatments with pure and hybrid Arkansas rice bran with cecal from **(A)** 2 weeks, **(B)** 4 weeks, and **(C)** 6 weeks chicken. ^a-c^Mean values denoted by the different letters within each treatment were significantly different at *P* < 0.05.

*Salmonella* Typhimurium survival in the presence and absence of pureline and hybrid LaKast rice bran using cecal contents from 2-, 4-, and 6-week chickens are shown in **Figures 2A,B,C**, respectively. There were significant differences in *S*. Typhimurium populations between initial and 48 h samples (*P* < 0.05). Moreover, LaKast rice bran pure and hybrid resulted in less bacterial survival compared to the control.

When ceca from 2-week-old chickens were used in these experiments, *S*. Typhimurium populations were reduced by 1.07 log CFU/ml in control sample after 24 h while pureline and hybrid LaKast rice bran reduced *S*. Typhimurium by 3.00 and 2.54 log CFU/ml, respectively (**Figure 2A**). After 48 h, the control treatment had a 2.58 log CFU/ml reduction while the LaKast pureline and hybrid rice bran resulted in greater reductions in *S*. Typhimurium with 6.22 and 6.01 log CFU/ml reductions (*P* < 0.05), respectively. For samples containing 4-week-old chicken ceca, the control yielded 1.15 and 2.70 log CFU/ml reductions after 24 and 48 h incubation, respectively, while LaKast pureline and hybrid resulted in 2.22 (24 h) and 5.72 (48 h) log CFU/ml reduction and 3.10 (24 h) and 4.78 (48 h) log CFU/ml reduction (*P* < 0.05), respectively. For samples containing 6-week-old chicken ceca, greater bacterial reduction activities were observed by LaKast pureline and hybrid compared to 2- and 4-week ceca. In the control sample, the *S*. Typhimurium counts were reduced to 3.92 and 2.32 log CFU/ml (3.29 and 4.90 log reduction) after 24 and 48 h, respectively (**Figure 2C**). The initial cell density of *S*. Typhimurium was 7.27 log CFU/ml and LaKast pureline bran resulted in a 6.47 log reduction after 24 h and no viable *S*. Typhimurium after 48 h (*P* < 0.05). When samples were treated with LaKast hybrid bran, a 5.17 and 6.58 log CFU/ml reduction was exhibited after 24 and 48 h, respectively (*P* < 0.05).

We next examined the bacterial composition, via microbiome sequencing, in anaerobic mixed culture samples containing 2-, 4-, and 6-week-old chicken ceca exposed to LaKast pureline and hybrid bran as well as control at various culture time points (0, 6, 12, 24, and 48 h incubation starting from the initial inoculation with cecal contents). During sequencing data analysis with QIIME, some data groups were removed due to low read numbers.

**FIGURE 3:**
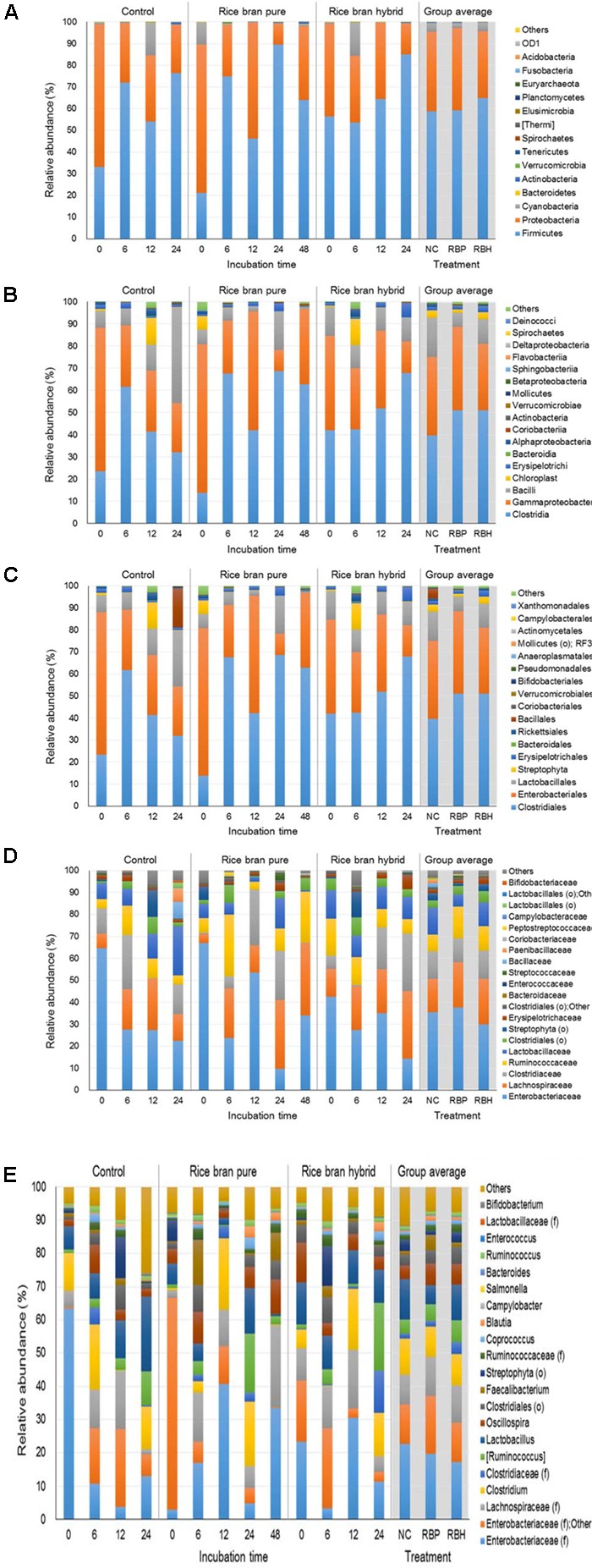
Relative abundance of major bacteria among different groups using ceca contents from 2 weeks of chicken broilers at a phylum **(A)**, class **(B)**, order **(C)**, family **(D)**, and genus level **(E)**.

**Figure 3** represents the major bacteria among groups identified in anaerobic cultures from phylum to genus level when ceca of 2-week-old chickens were used in the experiment. At the phylum level, *Firmicutes* and *Proteobacteria* were predominant, accounting for 60.76 and 35.37% of the entire phyla, respectively (**Figure 3A**). Along with incubation time, abundance of *Firmicutes* was increased in the control (33.13% at 0 h to 76.24% at 24 h) as well as LaKast pureline (21.12% at 0 h to 63.91% at 48 h) and hybrid (56.31% at 0 h to 85.09% at 24 h) while that of *Proteobacteria* was decreased [control: 65.73–22.52%, LaKast pureline (8.54–34.07%), and hybrid (42.85–14.31%)]. In the 24 h incubation samples, LaKast pureline yielded the lowest relative abundance of *Proteobacteria* with 9.71%, followed by LaKast hybrid with 14.31% and control with 22.56%. At the family level, *Enterobacteriaceae* abundance decreased with the incubation time; from 64.65% (initial) to 21.31% (24 h) in control, 66.96 % (initial) to 34.10% (48 h) in the LaKast pureline, and 34.01% (initial) to 14.25% (24 h) in the LaKast hybrid (**Figure 3D**). When 24 h samples of groups were compared, *Enterobacteriaceae* and *Lactobacillaceae* were predominant in the control group with 23.31 and 22.61%, respectively, while *Lachnospiraceae* and *Clostridiaceae* were predominant in the LaKast pureline incubation (31.34 and 22.34%) and hybrid (30.96 and 25.98%) groups. The genus level microbiome data showed a decrease of *Enterobacteriaceae*; the combined abundance of *Enterobacteriaceae* (family level) and Other was 63.94, 66.67, and 41.75% in the control, LaKast pureline, and LaKast hybrid, respectively, at the initial time point but they were reduced to 19.52, 9.53, and 14.05% after 24 h incubation (**Figure 3E**).

**FIGURE 4:**
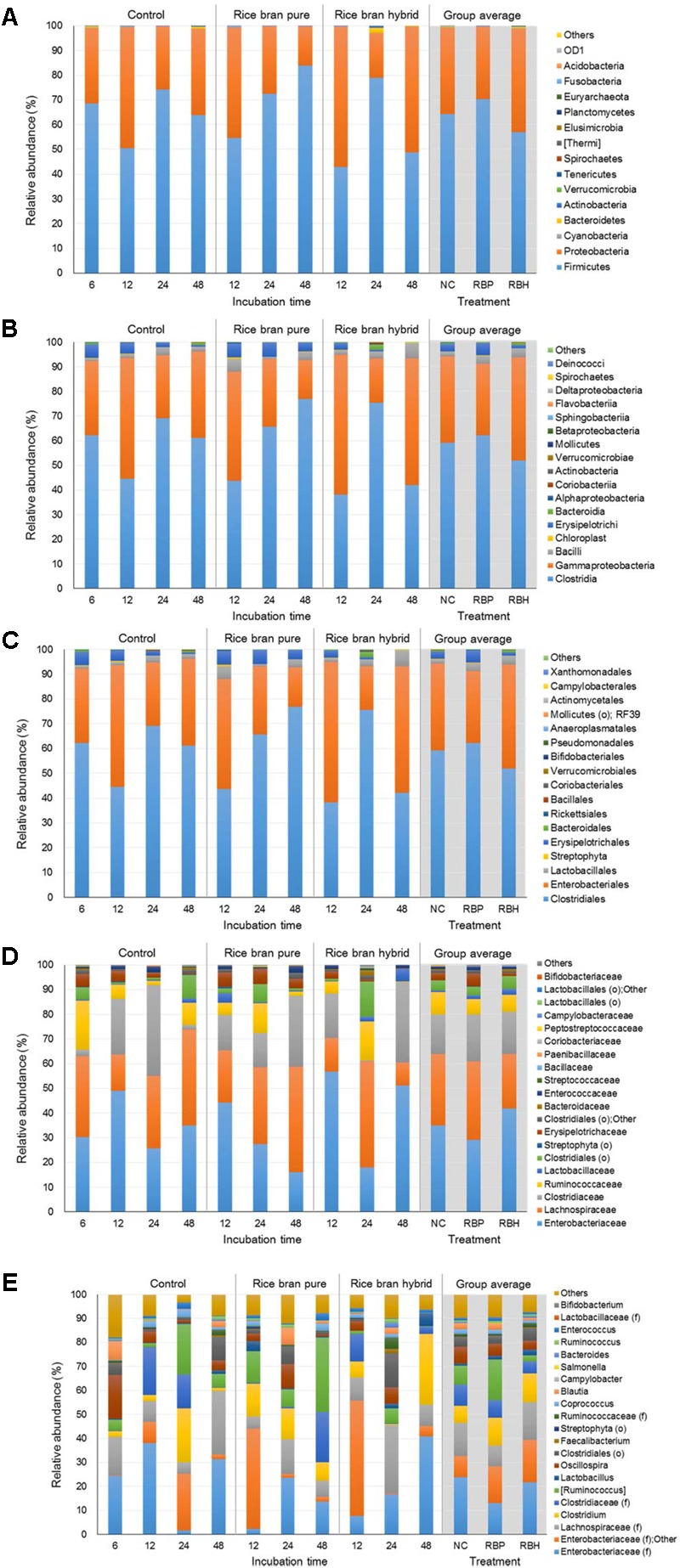
Relative abundance of major bacteria among different groups using ceca contents from 4 weeks of chicken broilers at a phylum **(A)**, class **(B)**, order **(C)**, family **(D)**, and genus level **(E)**.

**Figure 4** represents the major bacteria among groups identified in anaerobic cultures of control and LaKast treatments containing ceca of 4-week-old chickens from phylum to genus level. Similar to results from the experiment using 2-week-old chicken ceca, anaerobic cultures harbored the greatest proportion of *Firmicutes* constituting 63.82% of all phyla followed by *Proteobacteria* with 35.44% (**Figure 4A**). The *Proteobacteria* phylum includes a variety of pathogens such as *Campylobacter*, *Salmonella*, *E. coli*, *Vibrio*, and *Yersinia* (Nakagawa et al., 2007; Stopforth et al., 2007). In general, the proportion of *Proteobacteria* was reduced in the LaKast pureline from 44.65% (after 12 h) to 12.92% (after 48 h) while that of the control and the LaKast hybrid fluctuated during incubation times. *Enterobacteriaceae*, a large family of Gram-negative bacteria that contains pathogens such as *Salmonella*, *E. coli*, *Yersinia*, and *Shigella*, belongs to the *Proteobacteria*, and it exhibited a similar pattern at the family level with *Proteobacteria* at the phylum level (**Figure 4D**). The abundance of *Enterobacteriaceae* in LaKast pureline was reduced from 44.23% (12 h) to 15.88% (48 h). Comparing the bacterial abundance in 48 h samples at genus level, the lowest abundance of *Enterobacteriaceae* (family level) with Other was in LaKast pureline sample 15.60% while the control was 33.42% and LaKast hybrid was 45.13% (**Figure 4E**).

**FIGURE 5:**
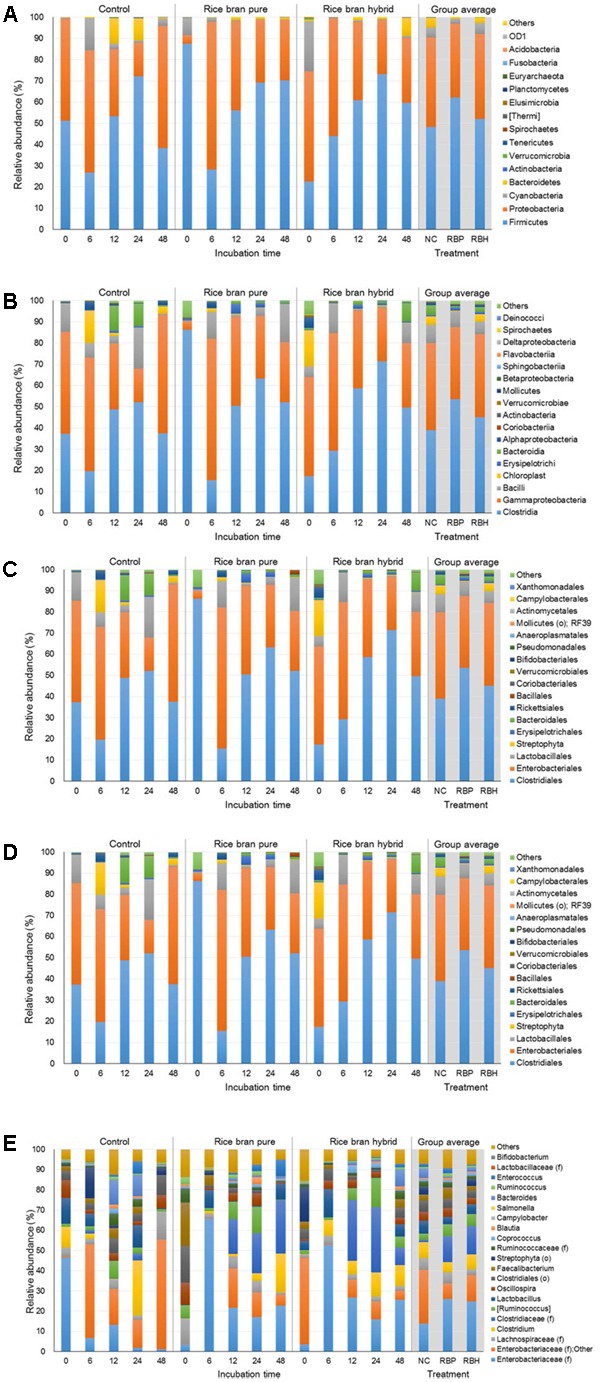
Relative abundance of major bacteria among different groups using ceca contents from 6 weeks of chicken broilers at a phylum **(A)**, class **(B)**, order **(C)**, family **(D)**, and genus level **(E)**.

**Figure 5** represents the major bacteria among groups identified in anaerobic cultures from phylum to genus level when ceca of 6-week-old chickens were used in the experiment. Similar to experiments using 2- and 4-week chickens, these incubations showed similar composition at the phylum level. The most predominant bacterial group was *Firmicutes* with 54.23% and *Proteobacteria* (39.03%; **Figure 5A**). In general, the proportion of *Proteobacteria* was reduced during incubation and LaKast pureline exhibited the lowest abundance (28.37%) of *Proteobacteria* at the end of the incubation (48 h) followed by LaKast hybrid (30.53%) and the control group (57.59%). This trend was also observed at other levels. LaKast pureline resulted in the lowest abundance of *Enterobacteriaceae* (28.30%, **Figure 5D**) at the family level and *Enterobacteriaceae* (family level) + Other (28.25%, **Figure 5E**) at the genus level while the control exhibited the highest proportion (55.53 and 55.39%, respectively).

In this study, *Salmonella* was artificially inoculated in anaerobic culture to examine the prebiotic effects of LaKast rice. *Salmonella* belong to the *Enterobacteriaceae* family and the *Proteobacteria* phylum. In 16s rRNA gene sequencing, a ≥ 97% sequence identity is considered a positive identification (Janda and Abbott, 2007; Reller et al., 2007). Indeed, *Salmonella* artificially inoculated in chicken carcass rinsates were identified as *Enterobacteriaceae* at the genus level (Kim et al., 2017b). Our previous study reported that chicken ceca without any prebiotic treatment were dominated by *Bacteriodetes* (52.21%) and *Firmicutes* (34.35%; Park et al., 2016) while abundance of *Proteobacteria* was relatively small with 5.91%. However, the present study indicates that *Proteobacteria* are the second most predominant bacteria in anaerobic cultures at the phylum level and this could be attributed to the *Salmonella* spiked into these experiments.

**FIGURE 6:**
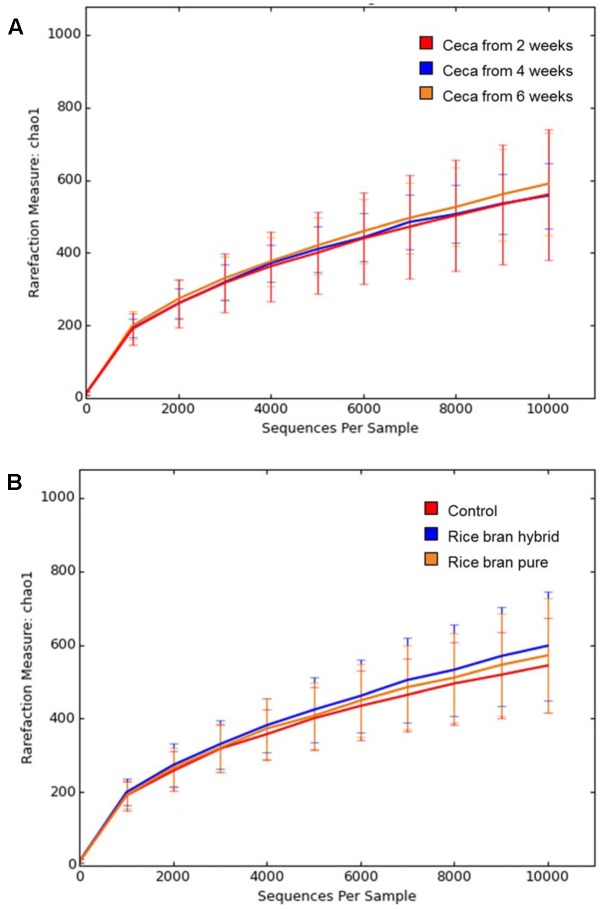
Rarefaction curves of Chao 1 in each group determined by **(A)** weeks of used ceca and **(B)** treatment (control or LaKast rice treated) from alpha diversity analysis.

The Chao 1 rarefaction plot which was employed to estimate species richness is presented in **Figure 6**. Anaerobic cultures containing cecal contents from older broilers exhibited more diverse microbiota. Sample groups with 6-week ceca showed the highest rarefaction value followed by groups with 4- and 2-week ceca (**Figure 6A**). Samples treated with LaKast rice bran exhibited greater diversity compared to the control groups, indicating that LaKast rice brans (both pureline and hybrid) resulted in richer microbiota than the control (**Figure 6B**). Gut microbiota play an important role to host health and commensal microbiota in the intestine can help to prevent colonization of invading pathogenic bacteria by interacting with pathogens directly and enhancement of defense mechanisms; thus diverse microbiota in this study would be expected to be more resistant to pathogens such as *Salmonella* (Kamada et al., 2013).

In our plating experiments, the population of *Salmonella* was approximately 7 log CFU/ml in the anaerobic culture at the initial time point, indicating that *Salmonella* was dominant in samples. However, no *Salmonella* was identified in the microbiome analysis. These results suggest that the spiked *Salmonella* may be identified as *Proteobacteria* at the phylum level and *Enterobacteriaceae* (family level) or “Other” in the microbiome analysis at the genus level. Therefore, lower abundance of *Proteobacteria* and *Enterobacteriaceae* in LaKast rice samples compared to control in all experiments using ceca from different chicken ages may be due to the significant reductions of *Salmonella* as shown in plating experiment results.

Arkansas is the top producer of rice in the United States. Thus, tremendous quantities of rice bran as a by-product are available (Talbert and Burgos, 2007; Wilson et al., 2007). The present study clearly demonstrated the beneficial functionality of LaKast rice bran to inhibit pathogenic bacteria in both plating and microbiome analysis. In addition, with reduction of *Salmonella*-related bacteria, LaKast rice treated samples exhibited more diverse microbiota. Rice bran is a natural product derived from rice and thus the use of rice bran or fractions of rice bran could be acceptable and favorable to both the consumer and the animal feed industry. The results suggest that adding rice bran or bran fractions to animal feed as a biological supplement can contribute to ensure poultry safety, especially when antibiotics in feed are likely to be prohibited in the future. In conclusion, the present study demonstrates that AR LaKast rice bran or fractions thereof can add economic value to rice bran, benefiting both the rice and the poultry feed industries in post-antibiotic feed agriculture.

## Author Contributions

SK, PR, SP, and SR designed the experiment. SK, PR, and SP performed the experiment and analyzed the data. SK wrote the manuscript, and PR, SP, and SR revised the manuscript.

## Conflict of Interest Statement

The authors declare that the research was conducted in the absence of any commercial or financial relationships that could be construed as a potential conflict of interest. The reviewer GS and handling Editor declared their shared affiliation.
